# Association between lactobacillus levels, depressive mood, and BMI in college students: the moderating role of physical activity

**DOI:** 10.3389/fnut.2025.1603169

**Published:** 2025-07-01

**Authors:** Youliang Wu, Qing Yi, Zihan Qi, Yao Yin, Yufei Qi

**Affiliations:** ^1^Hefei Institutes of Physical Science, Chinese Academy of Sciences, Hefei, China; ^2^University of Science and Technology of China, Hefei, China; ^3^Hefei Normal University, Hefei, China; ^4^Faculty of Sports and Exercise Science, Universiti Malaya, Kuala Lumpur, Malaysia; ^5^College of Traditional Chinese Medicine, Hunan University of Chinese Medicine, Changsha, China; ^6^Progression School of Upper Secondary, Beijing College of Finance and Commerce, Beijing, China; ^7^Department of Physical Education and Research, Central South University, Changsha, China

**Keywords:** Lactobacillus, depressive mood, physical activity, body mass index, university students

## Abstract

**Objectives:**

To investigate the association between Lactobacillus levels, depressive mood, and body mass index (BMI) among Chinese college students. In addition, to examine whether depressive mood mediates the association between Lactobacillus levels and BMI and whether physical activity (PA) moderates this association.

**Methods:**

This cross-sectional study recruited 423 Chinese college students using a multi-stage stratified sampling method. Lactobacillus levels were measured from stool samples, depressive mood was assessed using a well-designed depression scale, PA was tracked with accelerometers, and BMI was calculated using calibrated electronic scales. Confirmatory factor analysis, correlation analysis, regression analysis, and tests for mediation and moderation effects were conducted using SPSS and AMOS software.

**Results:**

Lactobacillus levels exhibited significant negative correlations with depressive moods (r = −0.131, *p* < 0.01) and BMI (r = −0.113, *p* < 0.05), while depressive mood showed a positive correlation with BMI (r = 0.117, *p* < 0.05). Mediation analysis revealed that depressive moods mediated the association between Lactobacillus levels and BMI, with an indirect effect of −0.021 (95% CI: −0.062 to −0.001). PA significantly moderated the association between Lactobacillus levels and BMI, as evidenced by an interaction coefficient of 0.009 (*p* < 0.001).

**Conclusion:**

Depressive mood could mediate the association between Lactobacillus levels and BMI, with PA playing a moderating role. This study provides new evidence for weight and depression management in college students.

## 1 Introduction

Obesity has emerged as a global health challenge, with its prevalence rising at an alarming rate (1). Obesity and overweight are major risk factors for various chronic diseases, including diabetes (2), cancer (3), cardiovascular diseases (4), and hypertension (5). BMI, defined as the ratio of weight (kg) to the square of height (m^2^), is a commonly used indicator for classifying overweight and obesity in adults. BMI categorizes overweight as 25–29.9 and obesity as ≥30 (6). In China, the adult obesity rate has reached 16.4% (7), higher than the global average (8). College students, burdened by academic pressure, insufficient PA, and prolonged sedentary behavior, also experience a notably high prevalence of obesity (9).

Growing evidence suggests that gut microbiota plays a critical role in the development of obesity (10), offering new possibilities for microbiota-mediated weight management interventions. Obesity is closely associated with changes in gut microbiota composition, such as alterations in the Bacteroidetes/Firmicutes ratio and levels of specific microbial genera (11). Probiotics, which are live microorganisms that enhance gut microbiota function, benefit the host, with Lactobacillus being the most prominent type (12). Currently, probiotic supplementation is gaining acceptance as an obesity intervention, with several reviews confirming its significant role in managing obesity (13, 14). Studies indicate that Lactobacillus can effectively alleviate obesity symptoms by reducing blood lipids, improving gut microbiota, regulating the immune system, and influencing metabolism (14–17). Notably, recent findings further reveal that gut microbiota-based interventions, including probiotics, synbiotics, and prebiotics, hold promising prospects for addressing diabetes (18), non-alcoholic fatty liver disease (19), and their related metabolic complications.

Beyond its role in host metabolism, the gut microbiota communicates with the central nervous system via the microbiota–gut–brain axis, thereby modulating mood and behavior (20, 21). Among them, depression is highly prevalent in college students and ranks as the fourth leading cause of suicide among individuals aged 15–29 (22). Research has indicated that the composition of gut microbiota may help mitigate depression (23), and such microorganisms are termed psychobiotics. Psychobiotics, primarily including Lactobacillus and Bifidobacterium, can produce neuroactive substances such as gamma-aminobutyric acid and serotonin (24). In rodent models, Lactobacillus has been shown to prevent postpartum depression via the microbiota-gut-brain axis (25). Additionally, Lactobacillus can alleviate depression-like symptoms caused by dendritic cytokine deficiencies (26).

Obesity and depression frequently co-occur and interact in a self-perpetuating cycle. The risk of depression increases by 55% when BMI ≥ 30 kg/m^2^, and the association between obesity and depression is even stronger when BMI ≥ 40 kg/m^2^ (27). Blasco et al. (28) conducted a longitudinal study on obesity and depression mood between 2012 and 2017, highlighting depression as a risk factor for obesity. Contemporary understanding of depression has evolved, recognizing weight gain and increased appetite as key “typical” core symptoms (29). A national survey of 43,093 U.S. adults, for instance, found that the prevalence of major depressive disorder with atypical features was nearly 40% higher than that of the disorder without atypical features (30). Depressed individuals often improve their mood by consuming high-fat, high-sugar “comfort foods” (29), a process that is linked to excessive activation of the hypothalamic–pituitary–adrenal axis, elevated cortisol levels, fat accumulation, and insulin resistance (31), all of which may contribute to weight gain. Therefore, effectively addressing depressive symptoms is expected to mitigate the obesity-promoting tendencies mentioned above.

Overall, Lactobacillus can directly reduce BMI and effectively alleviate depressive mood levels, and the latter, in turn, contributes to BMI reduction. Thus, it is plausible to hypothesize that depressive mood mediates the association between Lactobacillus levels and BMI. Additionally, existing evidence suggests that PA moderates this association. To our knowledge, no prior studies have examined whether depressive mood mediates the association between Lactobacillus levels and BMI, nor have they investigated whether PA moderates this association. Addressing this knowledge gap is essential, as the findings could provide preliminary empirical evidence on the mediating role of depressive mood and the moderating effect of PA on this association. Moreover, these findings provide a scientific foundation for designing targeted mental health and weight management interventions tailored to college students.

Therefore, this research conducted a cross-sectional study to investigate the association among Lactobacillus levels, depressive mood, and BMI in college students, as well as the moderating effect of PA on the association. This study aims to address three primary questions: (1) What is the association among Lactobacillus levels, depressive mood, and obesity in Chinese college students? (2) Does depressive mood mediate the association between Lactobacillus levels and BMI? (3) Does PA moderate the association between Lactobacillus levels and BMI?

## 2 Materials and methods

### 2.1 Study design and participants

**Figure 1:**
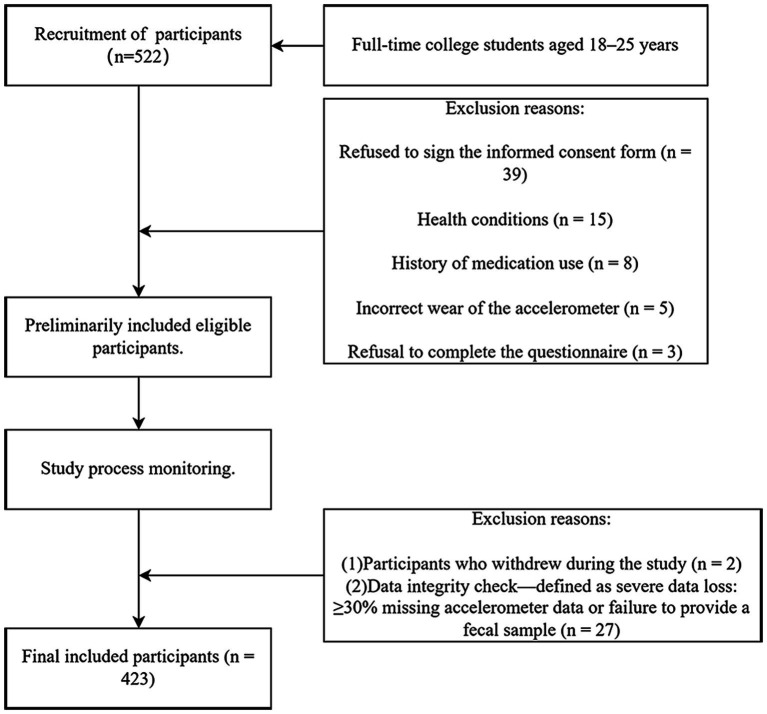
Participant selection flow chart.

A multi-stage stratified sampling method was employed to recruit college students from multiple universities, ensuring the diversity and representativeness of the sample. Inclusion criteria included full-time college students aged 18–25 years who could understand and voluntarily sign an informed consent form, had no severe chronic diseases (e.g., cardiovascular diseases or diabetes), no history of mental disorders (e.g., diagnosed depression or anxiety), and no use of probiotics or antibiotics in the past three months. Exclusion criteria encompassed inability to properly wear or use the research equipment (e.g., accelerometers); refusal to participate in the questionnaire survey or provide stool samples; withdrawal from the study, or severe data missing. Severe data missing was defined as a participant missing ≥30% of their accelerometer data or failing to provide a stool sample. Ultimately, 27 participants were excluded due to severe data loss. After rigorous screening (Figure 1), a total of 423 college students were included in this study. To improve participant compliance, the study provided a gift card incentive to each student who completed the data collection process. The study received approval from the Biomedical Ethics Committee of the Hefei Institutes of Physical Science, Chinese Academy of Sciences (SWYX-Y-2021-53).

### 2.2 Measurements

#### 2.2.1 Lactobacillus

Under professional supervision, students collected stool samples in sterile containers in the morning, which were promptly stored in low-temperature preservation boxes. The samples were swiftly transported to a specialized laboratory, where advanced molecular biological techniques, such as quantitative fluorescence PCR, were employed to accurately detect Lactobacillus using specific primers. Based on the results, Lactobacillus levels were categorized as abnormal (coded as 0) or normal (coded as 1). Based on the reference standard for healthy population microbiome research (32), this study classified a relative abundance of Lactobacillus below 1% as abnormal and ≥ 1% as normal. In subsequent sensitivity analyses, mediation models were re-estimated using the raw abundance values, and the core findings remained essentially unchanged (Supplementary materials 1, 2).

#### 2.2.2 Depressive mood

The depressive mood scale used in this study was developed based on Radloff’s CES-D scale (33) and the shortened version proposed by Kohout et al. (34). The scale is designed to measure depressive mood states over the past two weeks (e.g., sadness, hopelessness), rather than clinical depression. It contains 9 items, scored on a 4-point scale ranging from “never” to “always,” scored 1 to 4. Its Cronbach’s *α* coefficient was 0.944, indicating high internal consistency and reliability for measuring depressive mood traits. Confirmatory factor analysis (CFA) showed model fit indices as chi-square = 163.789 (df = 27), chi-square/df = 6.066 (*p* < 0.000), GFI = 0.912, AGFI = 0.854, CFI = 0.956, RMSEA = 0.110, and RMR = 0.036. According to SEM fit criteria (35), an RMSEA ≤ 0.08 is considered indicative of a good fit, while values between 0.08 and 0.12 fall within an acceptable range. In the present study, RMSEA was 0.110. Although this exceeds the ideal threshold, when taking into account the sample size and the short-form nature of the scale, this result remains within the acceptable range. Overall, it suggests that the scale demonstrates satisfactory structural validity.

#### 2.2.3 PA

Each participant was provided with a high-precision accelerometer (ActiGraph, Pensacola, Florida) and instructed on its use and precautions. Participants were required to wear the accelerometer on their waist continuously for 7 days, except during sleep, bathing, or other special circumstances, to ensure data integrity and accuracy. The accelerometer recorded acceleration data during daily activities, which was analyzed using specialized software (e.g., ActiGraph’s proprietary software) to calculate daily PA levels. The study primarily used the average daily total activity as the core indicator, derived by dividing the total weekly activity by 7.

#### 2.2.4 BMI

Body weight (accurate to 0.1 kg) and height (accurate to 0.01 m) were measured using rigorously calibrated electronic scales (brand: Omron, model: HBF-260 T1) and stadiometers (brand: Shanghe, model: SH-200G), respectively. BMI was calculated using the international standard formula: BMI = weight (kg) ÷ height^2^ (m^2^). BMI was used as a key indicator to assess obesity levels among college students.

### 2.3 Statistical methods

Data analysis was conducted using SPSS 26.0 and AMOS 24.0. Descriptive statistics were used to summarize basic characteristics. Bivariate correlation analysis was performed to examine interdimensional association and measure the strength of correlation between variables, with results expressed as mean ± standard deviation. Confirmatory factor analysis was performed on the scale using AMOS 24.0, evaluating structural validity with relevant fit indices. The indirect effect’s 95% confidence interval was estimated using the bias-corrected percentile method, with 5,000 bootstrap replications to minimize sampling bias (36). PA was added as a moderator in a moderation analysis. Interaction term coefficients were tested for significance to determine moderation, and the Johnson-Neyman method in PROCESS was applied to generate moderation effect plots. Unlike simple slope analysis, which evaluates the effect of the independent variable at selected moderator values, the Johnson–Neyman technique is appropriate when we want to determine comprehensively and precisely at which points across the moderator’s entire range the interaction effect is significant. Simple slope analysis only illustrates the IV–DV relationship at specific moderator values, whereas the Johnson–Neyman technique calculates the critical values of the moderator (e.g., PA) to identify the exact interval in which the main effect is significant.

## 3 Results

### 3.1 Demographic information

Independent samples t-tests were conducted to examine gender differences in Lactobacillus levels, BMI, depressive mood, and PA. Results indicated no significant gender difference in depressive mood (*p* = 0.612), whereas significant differences were identified for PA (*p* = 0.019) and Lactobacillus (*p* = 0.010), with girls outperforming boys in both. Significant gender differences were also observed in BMI, with boys outperforming girls (see Table 1).

**Table 1:** Independent samples t-test of different genders and dimensions.

Dimension	Mean equivalence t-test				Gender	Number of cases	M	SD
	T	DF	*P*	Mean Difference				
LAC	2.119	421	0.035	0.096	Girl	260	1.750	0.436
					Boy	163	1.650	0.478
BMI	−4.397	421	0.000	−0.307	Girl	260	2.0731	0.656
					Boy	163	2.3804	0.763
DEP					Girl	260	2.6705	0.806
	0.507	353.285	0.612	0.040	Boy	163	2.6305	0.778
PA	2.365	421	0.019	12.410	Girl	260	115.753	50.536
					Boy	163	103.344	55.584

LAC: Lactobacillus; DEP: depressive mood; DF: Degrees of Freedom; M: mean; SD: standard deviation.

### 3.2 Correlation analysis of variables

Pearson correlation analysis was conducted for all variables, as presented in Table 2. Lactobacillus was significantly positively correlated with depressive mood (r = 0.131, *p* < 0.01) and PA (r = 0.328, *p* < 0.01) but significantly negatively correlated with BMI (r = −0.113, *p* < 0.05). BMI showed a significant positive correlation with depressive mood (r = 0.117, *p* < 0.05), whereas both BMI and depressive mood exhibited non-significant negative correlations with PA (r = −0.069, *p* > 0.05; r = −0.039, *p* > 0.05).

**Table 2:** Correlation and descriptive statistics of variables in the predictive model.

Dimension	LAC	BMI	DEP	PA	M	SD
LAC	1	−0.113^*^	0.131^**^	0.328**	1.710	0.455
BMI		1	0.117^*^	−0.069	2.192	0.715
DEP			1	−0.039	2.655	0.794
PA				1	110.972	52.820

*p < 0.05, **p < 0.01.

### 3.3 Mediation effect of depressive mood

Model 4 of the SPSS PROCESS was utilized to examine the mediating effect of depressive mood in the association between Lactobacillus levels and BMI among college students. As shown in Table 3, after controlling for constants, Lactobacillus levels have a significant negative impact on depressive mood (*β* = −0.229, SE = 0.084, t = −2.713, *p* = 0.007). This indicates that higher levels of Lactobacillus are associated with lower levels of depressive mood. Lactobacillus levels also have a significant negative impact on BMI (β = −0.156, SE = 0.076, t = −2.037, *p* = 0.042), suggesting that higher levels of Lactobacillus are associated with lower BMI. Lactobacillus levels have a significant positive impact on BMI (β = 0.093, SE = 0.044, t = −2.103, *p* = 0.034), indicating that lower depressive mood is associated with lower obesity. In the mediation effect test, the total effect is −0.177 with a 95% CI (−0.327, −0.027), the direct effect is −0.156 with a 95% CI (−0.306, −0.005), and the indirect effect is −0.021 with a 95% CI (−0.062, −0.001), not including zero. These results suggest a significant mediating trend.

**Table 3:** Test of the mediating effect of DEP.

DV	IV	Unstd. Est		B	T	*P*	R^2^	95%CI	
		β	SE					LLCI	ULCI
DEP	Constant	3.047	0.149		20.406	0.000	0.017	2.753	3.340
	LAC	−0.229	0.084	−0.288	−2.713	0.007		−0.395	−0.063
BMI	Constant	2.210	0.189		11.691	0.000	0.023	1.839	2.582
	DEP	0.093	0.044	0.104	2.130	0.034		0.007	0.179
	LAC	−0.156	0.076	−0.218	−2.037	0.042		−0.306	−0.005

DEP mediation effect test value						
Effect	Effect	SE	T	*P*	LLCI	ULCI
Total effect	−0.177	0.076	−2.326	0.020	−0.327	−0.027
Direct effect	−0.156	0.076	−2.037	0.042	−0.306	−0.005
Indirect effect	−0.021	0.015			−0.055	−0.002

DV: dependent variable, IV: independent variable, CI: confidence interval; SE: standard error; LLCI: low limit for confidence interval; ULCI: upper limit for confidence interval.

### 3.4 Mediation analysis with covariates controlled

After controlling for covariates such as sex and grade level, the mediation analysis yielded clearer results. As shown in Table 4, the Lactobacillus (LAC) exerted a significant negative effect on DEP (*β* = −0.256, *p* = 0.003), representing a “net effect” after eliminating confounding factors, which authentically reflects their association. Grade level showed a significant positive effect on DEP (β = 0.093, *p* = 0.037), while the effect of sex was nonsignificant (*p* = 0.123). For BMI, both sex (β = 0.150, *p* = 0.035) and grade level (β = 0.114, *p* = 0.004) had significant positive effects. Without covariate adjustment, these factors could conflate the relationship between LAC and BMI, leading to biased estimates. Moreover, after covariate adjustment, the direct effects of LAC (*p* = 0.036) and DEP (*p* = 0.045) on BMI remained significant. Compared to the unadjusted model, this approach more accurately isolates the independent effects of each variable, providing a robust basis for dissecting their interrelationships.

**Table 4:** Mediation analysis with covariates controlled.

DV	IV	Unstd. Est		B	T	*P*	R^2^	95%CL	
		β	SE					LLCI	ULCI
DEP	Constant	2.885	0.187		15.457	0.000	0.032	2.518	3.252
	LAC	−0.256	0.085	−0.322	−3.018	0.003		−0.422	−0.089
	Sex	−0.123	0.080	−0.075	−1.545	0.123		−0.280	0.034
	Grade	0.093	0.044	0.101	2.093	0.037		0.006	0.180
BMI	Constant	1.868	0.208		8.976	0.000	0.055	1.459	2.277
	DEP	0.087	0.043	0.097	2.010	0.045		0.002	0.173
	LAC	−0.160	0.076	−0.224	−2.099	0.036		−0.310	0.010
	Sex	0.150	0.071	0.101	2.110	0.035		0.010	0.290
	Grade	0.114	0.040	0.138	2.879	0.004		0.036	0.192

### 3.5 Test of the moderating effect of PA using a mixed model

Utilizing SPSS PROCESS Model 5, this study conducted a mixed model analysis to investigate the association between Lactobacillus levels, depressive mood, and BMI. The results showed significant effects (Table 5): for depressive mood as the dependent variable, the intercept was 3.047 with a SE of 0.149, yielding a t-value of 20.406 and a highly significant *p*-value (<0.001). Lactobacillus levels were significantly inversely correlated with Lactobacillus (*β* = −0.229, SE = 0.084, t = −2.713, *p* = 0.007), suggesting that higher Lactobacillus levels are associated with lower depressive mood. For BMI, the intercept coefficient was 3.568 (SE = 0.281, t = 12.678, *p* < 0.001), with Lactobacillus levels positively correlated with BMI (*β* = 0.099, SE = 0.042, t = 2.355, *p* = 0.019), indicating that higher depressive mood is associated with higher BMI. Lactobacillus had a significant negative impact on BMI (β = −0.988, SE = 0.156, t = −6.317, *p* < 0.001), and PA also demonstrated a negative effect (*β* = −0.015, SE = 0.002, t = −6.229, *p* < 0.001). The interaction term *Lactobacillus x PA* positively influenced BMI (β = 0.009, SE = 0.001, t = 6.262, *p* < 0.001). These findings elucidate the significant roles of each variable and their interactions, offering a strong foundation for further exploration of these associations.

**Table 5:** Mixed model analysis.

DV	IV	Unstd. Est		T	*P*	R^2^	95% CI	
		β	SE				LLCI	ULCI
DEP	Constant	3.047	0.149	20.406	0.000	0.017	2.753	3.340
	LAC	−0.229	0.084	−2.713	0.007		−0.395	0.063
BMI	Constant	3.568	0.281	12.678	0.000	0.108	3.015	4.122
	DEP	0.099	0.042	2.355	0.019		0.016	0.181
	LAC	−0.988	0.156	−6.317	0.000		−1.295	−0.680
	PA	−0.015	0.002	−6.229	0.000		−0.020	−0.010
Lac × PA		0.009	0.001	6.262	0.000		0.006	0.012

### 3.6 Johnson-Neyman moderation effect model

The moderating effect of PA was tested using SPSS PROCESS Model 1. As shown in Table 6, the interaction term *Lactobacillus × PA* yielded a coefficient (β) of 0.009, with a SE of 0.001, a t-value of 6.182, and a *p*-value of 0.000. The 95% CI (0.006, 0.012) confirmed the significant effect, indicating a notable interaction between *Lactobacillus levels* and PA that impacts the dependent variable. The overall model explained 9.6% of the variance in the dependent variable (R^2^ = 0.096), with an *F*-value of 14.874 and a *p*-value of less than 0.001, demonstrating the significance of the entire model.

**Table 6:** Test of the moderating effect of PA.

DV	IV	β	SE	*t*	*p*	LLCI	ULCI	R^2^	*F*	R^2^-chng
BMI	Constant	2.123	0.035	60.686	0.000	2.054	2.191	0.096	14.874	0.082
	PA	0.000	0.001	−0.266	0.791	−0.001	0.001			
	LAC	−0.030	0.080	−0.380	0.704	−0.188	0.127			
	Lac × PA	0.009	0.001	6.182	0.000	0.006	0.012		38.213	

**Figure 2:**
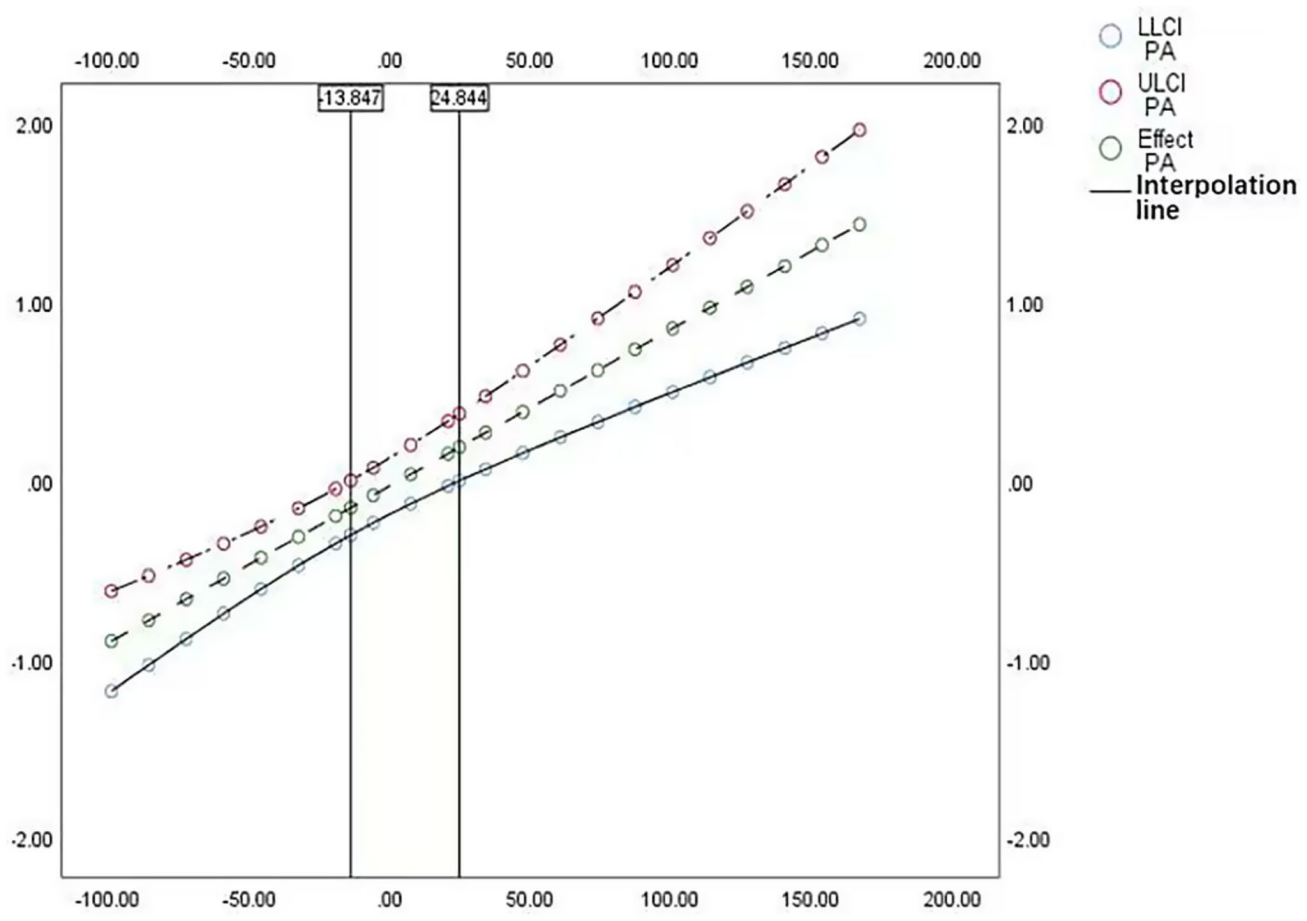
Johnson-Neyman plot illustrates the moderation model.

To better elucidate the moderating role of PA, we employed the Johnson-Neyman technique to identify the range of values where the moderation effect is significant. As shown in Figure 2, the moderation effect is statistically significant within the left interval below −13.847 and the right interval above 24.844. Specifically, when PA < −13.847 or PA > 24.844, the effect of Lactobacillus on BMI varies significantly as PA changes; between these two critical values, the moderation effect is not significant. Interpreting the moderation in terms of these thresholds and intervals provides a more intuitive understanding of how the effect behaves across the full range of the moderator, rather than relying solely on the interaction-term coefficient.

## 4 Discussion

### 4.1 Gender differences

From a gender perspective, our study revealed that females exhibited significantly higher Lactobacillus levels than males, which aligns with previous research. Previous studies suggest that gender influences gut microbiota composition, serving as a key determinant of microbial diversity and function (37). Our results highlight the importance of incorporating gender as a crucial variable in studies on the health effects of gut microbiota. In terms of BMI, males exhibited significantly lower levels than females, in line with established gender differences. This difference likely stems from gender-related variations in fat distribution and metabolic rates (38). These results provide additional evidence for the role of gender in weight management and underscore its implications for developing targeted interventions. Although our study found no significant gender differences in depressive mood, this does not diminish the role of gender in psychological health. However, other studies suggest that gender differences exist in the experience and expression of depressive mood (39). The absence of significant findings may stem from limitations such as sample size or measurement tools, highlighting the need for further investigation. In terms of physical activity, females were significantly more active than males, particularly in specific activity types, consistent with epidemiological evidence (40). This finding has important implications for designing gender-specific exercise interventions to enhance program effectiveness in promoting health across populations.

### 4.2 Correlations among variables

Among college students, the positive correlation between Lactobacillus levels and depressive mood reveals the intricate link between gut microbiota and mental health. This finding is consistent with existing evidence suggesting that the gut microbiome influences mood and behavior via the gut-brain axis (41). As a beneficial bacterium, the abundance of Lactobacillus in the gut may play a positive role in enhancing mental health (42). The observed positive association between Lactobacillus levels and PA offers new insights into the interplay between gut microbiota and lifestyle. This suggests that engaging in PA not only enhances physical health but may also exert a positive influence on mental well-being by fostering the proliferation of beneficial gut bacteria. The significant inverse correlation between Lactobacillus levels and BMI provides valuable insights into the role of gut microbiota in weight regulation. This finding suggests a potential role of Lactobacillus in regulating energy balance and fat metabolism, thereby contributing to weight maintenance.

### 4.3 Mediation effect of depressive mood

The study found a significant negative correlation between Lactobacillus levels and depressive mood, consistent with previous research (41). The prior research has indicated that the gut microbiota may influence mood and behavior through the gut-brain axis (41). Our results provide further evidence, suggesting that Lactobacillus may positively impact mental health by modulating the synthesis and release of neurotransmitters such as serotonin and dopamine. Additionally, Lactobacillus is believed to regulate the body’s response to stress, thereby potentially mitigating depression and anxiety (43). Another noteworthy finding is the significant negative correlation between Lactobacillus levels and BMI (44, 45), consistent with the previous research (46). Notably, the cross-sectional design of this study reveals only associations among Lactobacillus levels, BMI, and depressive mood, and cannot establish causality; therefore, these findings should be interpreted cautiously in clinical practice. Previous experimental studies have demonstrated that supplementation with psychobiotics (Lactobacillus) caneduce obesity (47) and depression (48) in adults. Lactobacillus supplementation may serve as a promising intervention to mitigate obesity and depression among college students. We also observed a significant positive relationship between depressive mood and BMI. As supported by existing literature, depressive mood may contribute to unhealthy eating behaviors, such as emotional eating, which in turn may influence BMI and waist circumference (49). This positive correlation suggests that depressive mood might lead individuals to engage in unhealthy eating behaviors, such as emotional eating. Our findings further substantiate this perspective, highlighting the critical role of mental health in weight management. Moreover, depressive mood might influence lifestyle choices, including overeating and physical inactivity, thereby affecting BMI (31).

Although the mediating effect of depressive mood between Lactobacillus levels and BMI was statistically significant, its effect size was small. This finding suggests that other factors, such as dietary habits (50), lifestyle (51), and genetic predispositions (52), may serve as additional mediators in this association. These factors could influence weight management by modulating the composition and functionality of the gut microbiota. Furthermore, the results revealed a significant negative effect of Lactobacillus on BMI, potentially attributable to its regulatory influence on the gut environment. Lactobacillus may affect energy absorption and metabolism by fostering the growth of beneficial bacteria while suppressing harmful ones, thereby influencing BMI.

### 4.4 Moderating role of PA

The significant moderating effect of physical activity (PA) on the association between Lactobacillus levels and BMI highlights the crucial role of PA in modulating the relationship between gut microbiota and health outcomes (53, 54). PA may enhance the growth and activity of beneficial bacteria, such as Lactobacillus, by improving blood circulation, promoting intestinal motility, and optimizing the gut environment. Mixed model analyses revealed negative coefficients for both Lactobacillus and PA, indicating their independent associations with lower BMI. However, the interaction term between Lactobacillus and PA showed a positive coefficient for BMI, suggesting that higher PA levels may weaken the effect of Lactobacillus in reducing BMI. That is, the BMI-lowering effect of Lactobacillus appeared less pronounced among individuals with high PA levels compared to those with lower PA levels. This suggests that the interaction between Lactobacillus and PA is non-additive in nature. At higher levels of PA, other factors—such as different microbial populations, dietary habits, or genetic influences—may exert a more dominant impact on BMI regulation. Previous research has shown that PA, particularly when combined with dietary interventions, can induce substantial changes in gut microbiota composition, including increased Lactobacillus abundance. These microbial shifts are strongly associated with weight loss and BMI reduction (55).

The Johnson-Neyman technique was employed to examine in detail the moderating effect of physical activity (PA) on the association between Lactobacillus levels and BMI. The results indicated that this moderating effect was particularly significant when PA levels were below −13.847 or above 24.844, highlighting the nuanced role of PA in the Lactobacillus-BMI relationship across different activity levels. Notably, at higher levels of PA, the inverse association between Lactobacillus and BMI was more pronounced, suggesting that PA may enhance the potential benefits of Lactobacillus in weight management. However, the moderating effect identified in this study remains correlational in nature, precluding causal inference. Encouragingly, previous experimental and longitudinal studies have shown that probiotic supplementation combined with PA can produce synergistic effects in reducing BMI (56). Such combined intervention strategies present promising approaches for clinical application. Overall, in evaluating the role of gut microbiota in weight regulation, our study underscores the importance of increasing PA levels and offers valuable insights for future research and intervention strategies.

### 4.5 Strengths and limitations

Regarding measurement tools, a key advantage of this study is the application of a high-precision accelerometer (ActiGraph, Pensacola, Florida) for objectively measuring PA, thus eliminating the recall bias typically associated with self-reported questionnaires. Additionally, the study utilized the validated 9-item short version of the CES-D (*α* = 0.944), which provides superior measurement accuracy relative to non-standardized scales. Methodologically, the study applied advanced statistical techniques, including mixed models and the Johnson-Neyman method, which improved the precision and robustness of the results. Additionally, by integrating Lactobacillus levels, depressive mood, PA, and BMI, the study established a multivariate analytical framework, facilitating a deeper comprehension of the intricate interrelations among these variables.

This study has four notable limitations. First, the cross-sectional design constrains the ability to establish causal relationships. Future research should adopt longitudinal designs to provide deeper insights into the dynamic relationships among these variables. Second, the inherent limitations of self-reported measures cannot be disregarded, as participant subjectivity may compromise data reliability. To enhance measurement precision, future studies should incorporate objective assessments, such as physiological monitoring and biomarker analysis. Third, this study did not comprehensively account for environmental factors that may influence gut microbiota and BMI, including campus environment, seasonal variations, and socioeconomic status. The omission of these variables may have introduced confounding effects. Therefore, future studies should be conducted under more rigorously controlled conditions to mitigate the influence of these potential confounders. Lastly, although the model captures the key relationships among variables, the R^2^ value of 0.096 indicates that only 9.6% of the variance in BMI is explained, potentially due to unmeasured confounders such as dietary patterns, sleep quality, and stress levels. Future research may benefit from the dynamic collection of multidimensional behavioral data or from implementing machine learning approaches to uncover potential nonlinear associations. Even when R^2^ values are relatively low (e.g., 0.1–0.5), a model can still be considered meaningful if key predictors reach statistical significance (57). This study emphasizes theoretical validation over predictive accuracy, and the value of the model is supported by significant mediation and moderation effects.

## 5 Conclusion

This study utilized a cross-sectional design with 423 college students to investigate the association among Lactobacillus levels, depressive mood, PA, and BMI. The results demonstrated that Lactobacillus levels were significantly negatively correlated with both depressive mood and BMI. Depressive mood served as a mediator in the relationship between Lactobacillus levels and BMI, while PA significantly moderated the effect of Lactobacillus on BMI. These findings revealed that depressive mood could mediate the association between Lactobacillus levels and BMI, and underscores the critical moderating role of PA. This study offers new insights into how PA modulates the association between Lactobacillus levels and BMI, providing valuable implications for targeted weight management and mental health interventions among college students.

## Data Availability

The raw data supporting the conclusions of this article will be made available by the authors, without undue reservation.
