# Supplementary material for: Association between lactobacillus levels, depressive mood, and BMI in college students: the moderating role of physical activity
Source: Front Nutr. 2025 Jul 1;12:1603169. doi: 10.3389/fnut.2025.1603169 (PMC12259426; doi:10.3389/fnut.2025.1603169)
Supplement: Supplementary file 1 [file Table_1.DOCX]

**Supplementary material 1: Testing the mediating effect of DEP**

| DV | IV | | Unstd.Est | | B | T | P | R^2^ | 95%CL | |
| --- | --- | --- | --- | --- | --- | --- | --- | --- | --- | --- |
|  |  |  | β | SE |  |  |  |  | LLCI | ULCI |
| DEP | Constant | | 2.824 | 0.084 |  | 33.506 | 0.000 | 0.012 | 2.658 | 2.990 |
|  | LAC | | -0.124 | 0.055 | -0.109 | -2.251 | 0.025 |  | -0.233 | -0.016 |
| BMI | Constant | | 2.109 | 0.144 |  | 14.624 | 0.000 | 0.027 | 1.839 | 2.582 |
|  | DEP | | 0.093 | 0.044 | 0.104 | 2.142 | 0.033 |  | 0.008 | 0.179 |
|  | LAC | | -0.121 | 0.050 | -0.118 | -2.447 | 0.015 |  | -0.219 | -0.024 |
|  | | DEP mediation effect test value | | | | | | | | |
| Effect | | | Effect | SE | t | p | | LLCI | ULCI | |
| Total effect | | | -0.133 | 0.050 | -2.686 | 0.008 | | -0.230 | -0.036 | |
| Direct effect | | | -0.121 | 0.050 | -2.447 | 0.015 | | -0.219 | -0.024 | |
| Indirect effect | | | -0.012 | 0.009 |  |  | | -0.032 | 0.001 | |
