# Supplementary material for: Association between lactobacillus levels, depressive mood, and BMI in college students: the moderating role of physical activity
Source: Front Nutr. 2025 Jul 1;12:1603169. doi: 10.3389/fnut.2025.1603169 (PMC12259426; doi:10.3389/fnut.2025.1603169)
Supplement: Supplementary file 2 [file Table_2.DOCX]

**Supplementary material 2 Mixed‐effects model analysis**

| DV | IV | Unstd.Est | | T | P | R^2^ | 95% CI | |
| --- | --- | --- | --- | --- | --- | --- | --- | --- |
|  |  | β | SE |  |  |  | LLCI | ULCI |
| DEP | Constant | 2.824 | 0.084 | 33.506 | 0.000 | 0.012 | 2.658 | 3.990 |
|  | LAC | -0.124 | 0.055 | -2.251 | 0.025 |  | -0.233 | 0.016 |
| BMI | Constant | 2.647 | 0.191 | 13.839 | 0.000 | 0.070 | 2.271 | 3.023 |
|  | DEP | 0.094 | 0.043 | 2.204 | 0.028 |  | 0.010 | 0.178 |
|  | LAC | -0.508 | 0.105 | -4.834 | 0.000 |  | -0.714 | -0.301 |
|  | PA | -0.006 | 0.001 | -4.180 | 0.000 |  | -0.008 | -0.003 |
| **Lac X Pa** | | **0.004** | **0.001** | **4.265** | **0.000** |  | **0.002** | **0.006** |
