# Supplementary material for: Association between lactobacillus levels, depressive mood, and BMI in college students: the moderating role of physical activity
Source: Front Nutr. 2025 Jul 1;12:1603169. doi: 10.3389/fnut.2025.1603169 (PMC12259426; doi:10.3389/fnut.2025.1603169)
Supplement: Supplementary file 3 [file Table_3.DOCX]

**Supplementary material 3 Collinearity diagnostics results**

| DV | IV | Unstandardized coefficient | | Standardized coefficient | t | VIF | R^2^ |
| --- | --- | --- | --- | --- | --- | --- | --- |
|  |  | β | SE | Beta |  |  |  |
| BMI | (Constant term) | 2.232 | 0.192 |  | 11.643 |  | 0.024 |
|  | LAC | -0.137 | 0.081 | -0.087 | -1.694 | 1.138 |  |
|  | DEP | 0.093 | 0.044 | 0.104 | 2.132 | 1.017 |  |
|  | pa | 0.000 | 0.001 | -0.036 | -0.706 | 1.121 |  |
